# Inhibition of astrocytic activity alleviates sequela in acute stages of intracerebral hemorrhage

**DOI:** 10.18632/oncotarget.22022

**Published:** 2017-10-24

**Authors:** Cheng-Di Chiu, Nai-Wei Yao, Jeng-Hung Guo, Chiung-Chyi Shen, Hsu-Tung Lee, You-Pen Chiu, Hui-Ru Ji, Xianxiu Chen, Chun-Chung Chen, Chen Chang

**Affiliations:** ^1^ School of Medicine, China Medical University, Taichung, Taiwan; ^2^ Institute of Biomedical Sciences, Academic Sinica, Taipei, Taiwan; ^3^ Graduate Institute of Biomedical Science, China Medical University, Taichung, Taiwan; ^4^ Department of Neurosurgery, China Medical University Hospital, Taichung, Taiwan; ^5^ Stroke Center, China Medical University Hospital, Taichung, Taiwan; ^6^ Department of Minimally Invasive Skull Base Neurosurgery, Neurological Institute, Taichung Veterans General Hospital, Taichung, Taiwan; ^7^ Department of Neurosurgical Oncology, Neurological Institute, Taichung Veterans General Hospital, Taichung, Taiwan

**Keywords:** acute stroke, intracerebral hemorrhage, astrocyte, blood–brain barrier, MRI

## Abstract

Neurological deterioration of intracerebral hemorrhage (ICH) mostly occurs within the first 24 hours. Together with the microglia/macrophages (MMΦ), astrocytes are important cell population responsible for many brain injuries but rarely being highlighted in acute stage of ICH. In present study, we induced rats ICH either by collagenase or autologous blood injection. Experimental groups were classified as vehicle or Ethyl-1-(4-(2,3,3-trichloroacrylamide)phenyl)-5-(trifluoromethyl)-1H-pyrazole-4-carboxylate (Pyr3) treatment group (*n* = 9, each group). MRI assessments after ICH were used to evaluate the hematoma progression and blood–brain barrier (BBB) integrity. The glia cells accumulations were examined by GFAP and Iba1 immunohistochemistry, respectively. Abundant astrocytes but few MMΦ were observed in hyperacute and acute ICH. Upon suppression of astrocyte activity, ICH rats exhibited decreased size of hematoma expansion, less BBB destruction, reduced astrocyte accumulation in perihematomal regions, postponed course of hemoresolution and gain better outcomes. These finding provide evidence that activated astrocytes are crucial cell populations in hyperacute and acute ICH, and their modulation may offer opportunities for novel therapy and patient management.

## INTRODUCTION

Most neurological deterioration of intracerebral hemorrhage (ICH) occurs within the first 24 h and is defined as either hyperacute (within 3 h) or acute (within 24 h) stages of ICH [1, 2]. Despite the high mortality, no definitive, effective medical treatment is available for ICH, and the efficacy of neurosurgical intervention for hyperacute or acute ICH remains controversial [3]. Therefore, understanding the pathophysiology during the first 24 h, particularly the temporal changes of hematoma, may be crucial in identifying an optimal therapeutic time window for ICH management.

From the initial hours of onset to the first few days after ICH onset, inflammatory responses are a critical concern, which are dominated by not only the resident astrocytes and microglia but also the peripheral neutrophils and macrophages [4, 5]. Among these cells, the reactive astrocytes accumulate in the perihematomal region as early as 1–3 days after ICH onset, which is much earlier than the accumulation of microglia and macrophages (3–7 days after ICH) [6]. Nevertheless, the role of astrocytes within the first 24 h of ICH has not been fully addressed.

Recently, reactive astrocytes are considered as a “double-edged sword” to have dual roles in several types of CNS injuries including ICH [7, 8]. The beneficial effects could be provided by tissue repair and cell protection [6, 9, 10], while potentially harmful effects might be caused by induction of proinflammatory responses [11] and oxidative stress [12]. They are believed to be directly or indirectly related to the BBB integrity, cerebral edema, release of chemokines and cytokines, and the resorption of hematoma [10, 13-17]. There have been calls for studies to meticulously determine the significance of astrocytes within the first 24 h of ICH.

Manipulating astrocytic activity is a common approach to investigating the role of astrocytes in different types of central nervous system (CNS) diseases [18]. Thrombin is a blood-derived factor that permeates the brain during hemorrhage and can upregulate transient receptor potential canonical 3 (TRPC3, a nonselective calcium channel), resulting in excessive astrocyte activation [19, 20]. Pyr3, a specific TRPC3 inhibitor, can suppress astrocytic activity and attenuate astrogliosis in ICH mice [21]. In this study, we used Pyr3 to modulate the activity of astrocytes in order to understand their roles during the first 24 h of ICH.

Magnetic resonance imaging (MRI) is an ideal method with high sensitivity for characterizing the temporal and spatial evolution of hematoma following ICH [22-24]. We first characterized the morphological changes of hematoma and BBB integrity in the hyperacute and acute ICH by using multiparametric MRI methods. Second, we investigated whether astrocytes are a vital cell population in these stages through immunohistochemistry. Finally, by using Pyr3, we demonstrated the critical role of astrocytes in hematoma formation, hemorrhage resolution, and BBB destruction. Our findings indicate that activated astrocytes are crucial for the pathogenesis of ICH during the hyperacute and acute stages.

## RESULTS

### Characterization of collagenase-ICH and within 24 h through MRI

**Figure 1:**
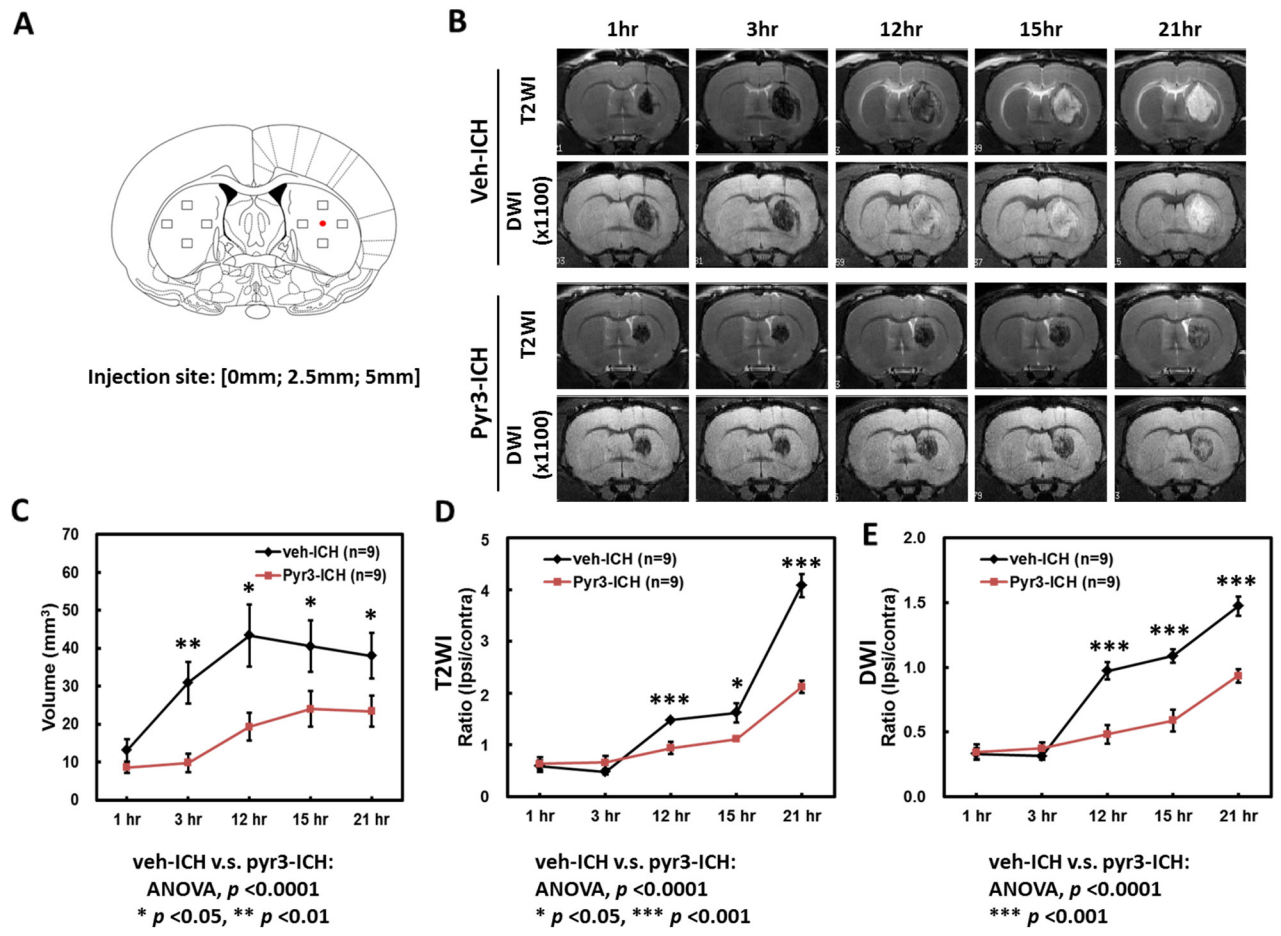
Temporal evolution of hematoma over the first 24 h of ICH **(A)** The injection site and the preselected regions of the striatum for histological quantification were represented by the red dot and boxes, respectively. **(B)** Representative T2WI and DWI images of vehicle- and Pyr3-treated rats were shown. The temporal evolution of hematoma in both groups was monitored through MRI at 1, 3, 12, 15, and 21 h after ICH induction. **(C)** Volumes of the hematomas at different time points were estimated using T2WIs (*n* = 9 per group). **(D)** The hematoma of the Pyr3-treated group shows a significantly lower signal ratio in T2WI during the first 24 h. **(E)** A significant decrease in signal ratio in DWI is also observed between groups. Data are presented as the mean ± SEM. ^*^p < 0.05; ^**^p < 0.01; ^***^p < 0.001.

Figure 1B presents representative T2-weight images (T2WI) and diffusion-weight images (DWI) images from the vehicle- and Pyr3-treated ICH rats. The temporal evolution of hematoma within 24 h after ICH induction on T2WI and DWI images is depicted in the upper rows of Figure 1B. The hematoma on both the T2WI and DWI images was initially hypointense with an isointense core at 1 and 3 h, and it became heterogeneous at 12 and 15 h compared with earlier time points. The lesion became uniformly hyperintense on both the T2WI and DWI images at 21 h, indicating that a progressive process of hemoresolution occurs within 24 h after ICH induction. Compared with the hematoma in the vehicle-treated group, Pyr3 treatment yielded hematomas that remained hypointense on both the T2WI and DWI images (Figure 1B, lower rows). The ratios of T2WI and DWI signals were quantified and plotted, as shown in Figure 1D and 1E, respectively. Significantly lower T2WI signal ratios were detected in the hematoma in the Pyr3-treated group at 12, 15, and 21 h after ICH induction (Figure 1D; *p* < 0.05 at 15 h and *p* < 0.001 at 12 and 21 h). The DWI signal ratios were significantly lower in the Pyr3-treated group at 12, 15, and 21 h after ICH induction (Figure 1E; all *p* < 0.001 for vehicle-treated vs. Pyr3-treated groups).

In addition, in the vehicle group, the size of the hematoma was significantly increased at 3 h after ICH induction (Figure 1B and 1C, Student’s *t* tests: all *p* < 0.05 for 1 h vs. any other time point). Pyr3 treatment considerably reduced the size of the hematoma in the T2WI measurements at 3, 12, 15, and 21 h after ICH induction (ANOVA: F(1,80) = 49.134, *p* < 0.0001; Fisher’s post hoc tests: *p* < 0.001 at 3 h, and all *p* < 0.01 at 12, 15, and 21 h). These results suggest that the suppression of astroglial responses not only postpones the progress of hemoresolution but also reduces the size of the hematoma.

### Key role of astrocytes in hyperacute and acute ICH

**Figure 2:**
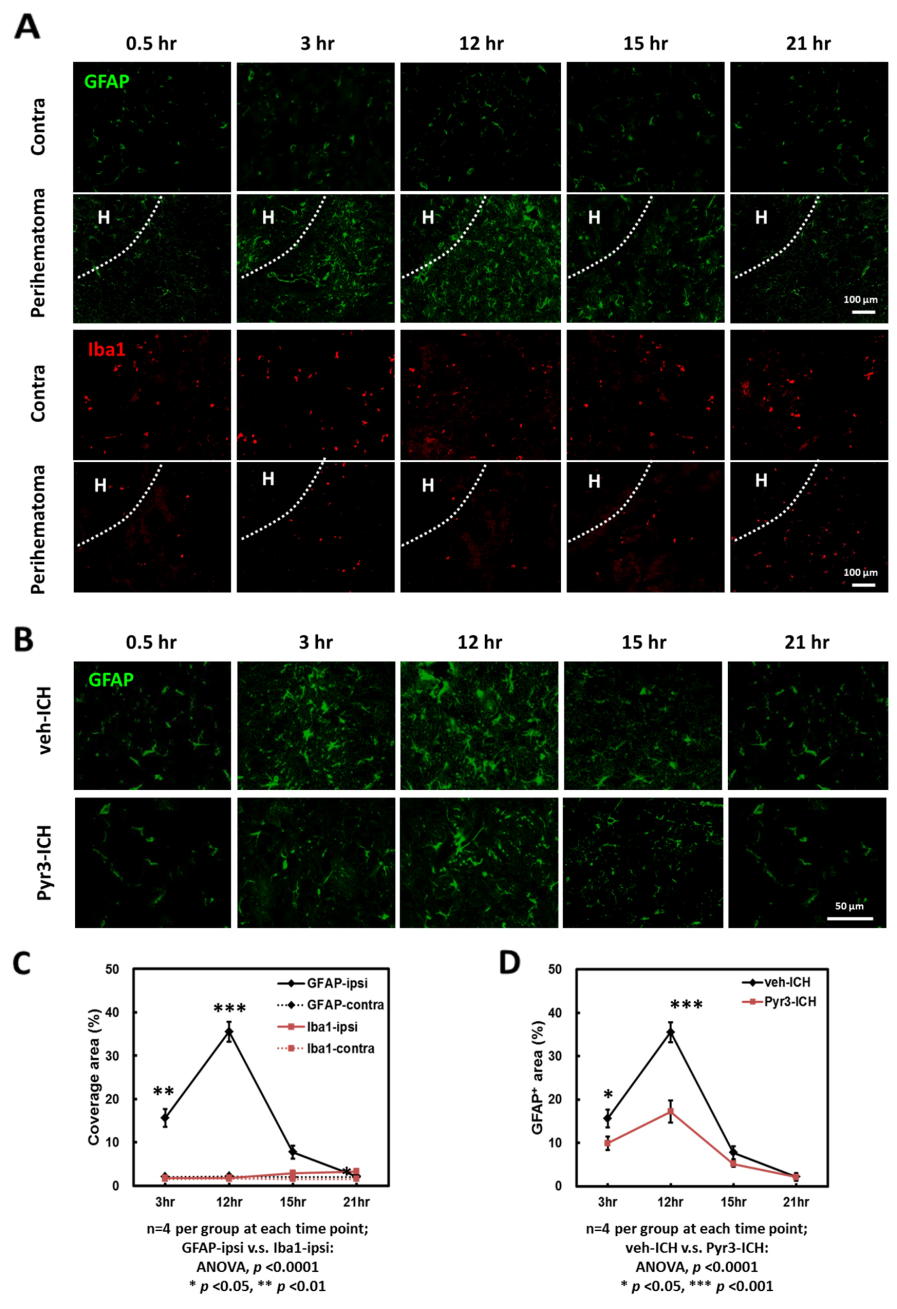
Time-dependent astrocyte accumulation in the perihematomal area within 24 h after ICH induction **(A)** Representative images of GFAP and Iba1 staining of ICH rats. The hematoma was indicated as “H”. **(B)** Representative GFAP staining of vehicle- and Pyr3-treated rats. **(C)** GFAP and Iba1 staining plot. **(D)** GFAP staining of vehicle- and Pyr3-treated groups plot. Quantification was performed by calculating the coverage of positive signals in four images captured from each of the four rats examined per group. Data are presented as the mean ± SEM. ^*^p < 0.05; ^**^p < 0.01; ^***^p < 0.001.

The astroglial, microglial/macrophagic responses to injuries were assessed at multiple time points within 24 h after collagenase-induced ICH (Figure 2). Figure 2A shows the representative GFAP staining for astrocytes (upper rows) and Iba1 staining for MMΦ (lower rows) in lesion and contralateral sites, respectively. The representative GFAP and Iba1 staining at 0.5 h in both groups was shown that there was no significant difference among contralateral and lesions sites (the first columns of Figure 2A and 2B). The immune-reactive areas of GFAP and Iba1 were quantified and plotted (Figure 2C). The GFAP-positive astrocytes mainly accumulated in the perihematomal region (Figure 2A and 2C). In the perihematomal regions, these astrocytes were observed at 3 h, and peaked at 12 h after ICH induction (Figure 2C, Student’s *t* tests: *p* < 0.01 for 3 vs. 12 h). The number of GFAP-positive cells then started to decrease at 15 and 21 h after stroke (Student’s *t* tests: *p* < 0.001 for 12 vs. 15 h, *p* < 0.001 for 12 vs. 21 h). Compared with GFAP staining, significantly fewer Iba1-positive MMΦwere observed in the perihematomal regions at all time points (Figure 2A and 2C; ANOVA: *p* < 0.0001 for GFAP vs. Iba1 staining). These findings suggest that astrocytes, rather than microglia or macrophages, play a more crucial role in pathogenic development in the hyperacute and acute stages of ICH.

### Reduction in perihematomal accumulation of astrocytes in Pyr3-treated ICH rats

The perihematomal accumulation of astrocytes in the vehicle- and Pyr3-treated ICH rats at 0.5, 3, 12, 15, and 21 h after ICH induction was further examined using GFAP staining (Figure 2B). The GFAP-positive areas were quantified and plotted (Figure 2D). The number of GFAP-positive astrocytes in the perihematomal area in the Pyr3-treated ICH rats decreased significantly throughout the course of the experiment compared with the vehicle-treated ICH rats (Figure 2D; ANOVA: F(1,24) = 14.113, *p* < 0.001; Fisher’s post hoc tests: all *p* < 0.0001 at 3 and 12 h after ICH induction). These results suggest Pyr3 decreases the pathologically activated astrocytes and may be closely associated with the evolution of ICH in the initial hours after onset.

### Blood-brain barrier (BBB) integrity of collagenase-induced ICH rats

**Figure 3:**
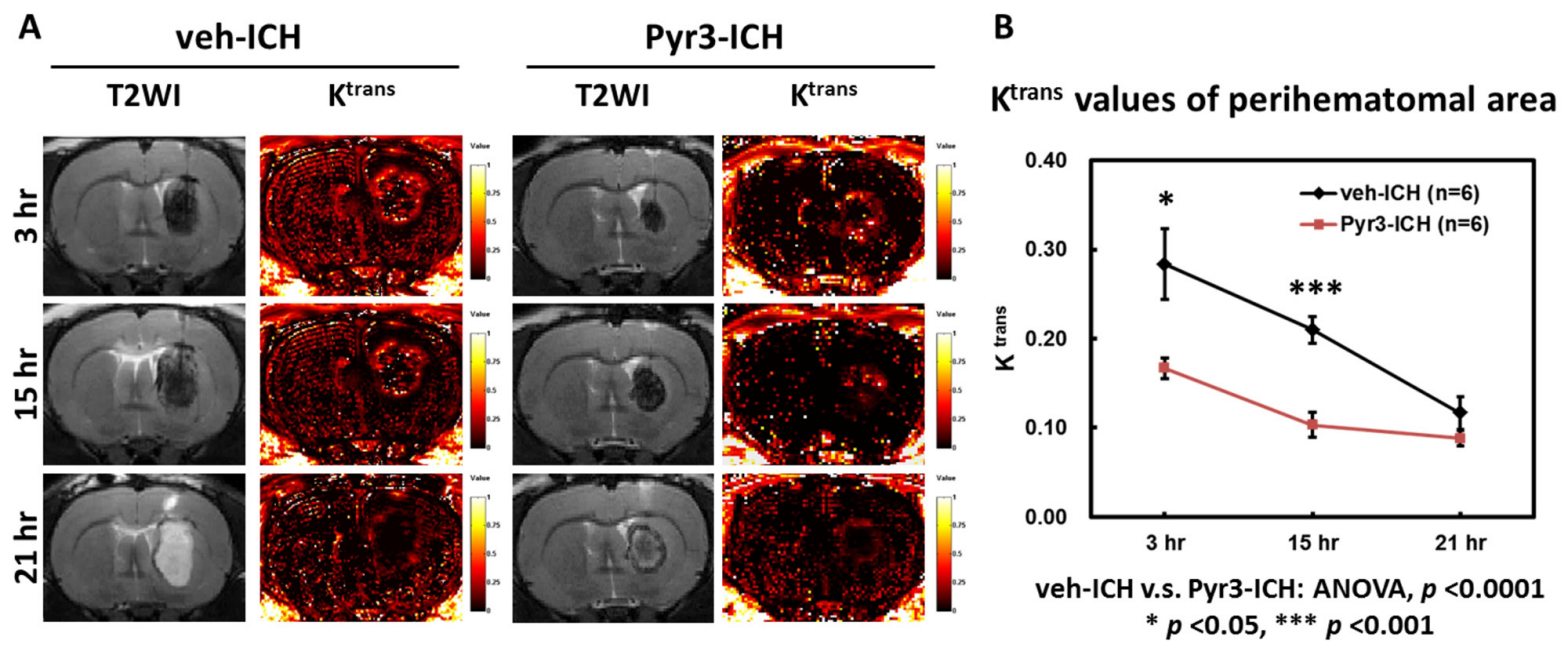
Pyr3 treatment significantly reduced BBB disruption in hyperacute and acute ICH **(A)** Representative MR images of vehicle- and Pyr3-treated rats. The color ranges from black (0/min) to orange (0.5/min) and white (1/min). **(B)** The graph of the K^trans^ values. Data are presented as the mean ± SEM. ^*^p < 0.05; ^**^p < 0.01; ^***^p < 0.001.

The temporal evolution of BBB integrity of the hematomas at 3, 15, and 21 h after ICH induction is illustrated in Figure 3. The representative T2WI and K^trans^ maps in the vehicle- and Pyr3-treated groups are shown in Figure 3A. The degree of BBB disruption was peaked at 3 h and subsequently decreased over time (Figure 3A, left column). Notably, Pyr3 treatment considerably reduced the K^trans^ signals in the Pyr3-treated groups at both 3 and 15 h after ICH induction compared with the vehicle-treated groups (Figure 3B; ANOVA: F(1,30) = 25.128, *p* < 0.0001; Fisher’s post hoc tests: *p* < 0.05 at 3 h and *p* < 0.001 at 15 h after ICH induction). No significant between-groups difference was observed at 21 h after ICH induction. These data may suggest that the suppression of astrocyte activity could reduce the BBB damage after ICH onset, particularly in the initial hours after onset.

### Pyr3-treated ICH rats exhibited better performance in neurobehavioral function tests

**Figure 4:**
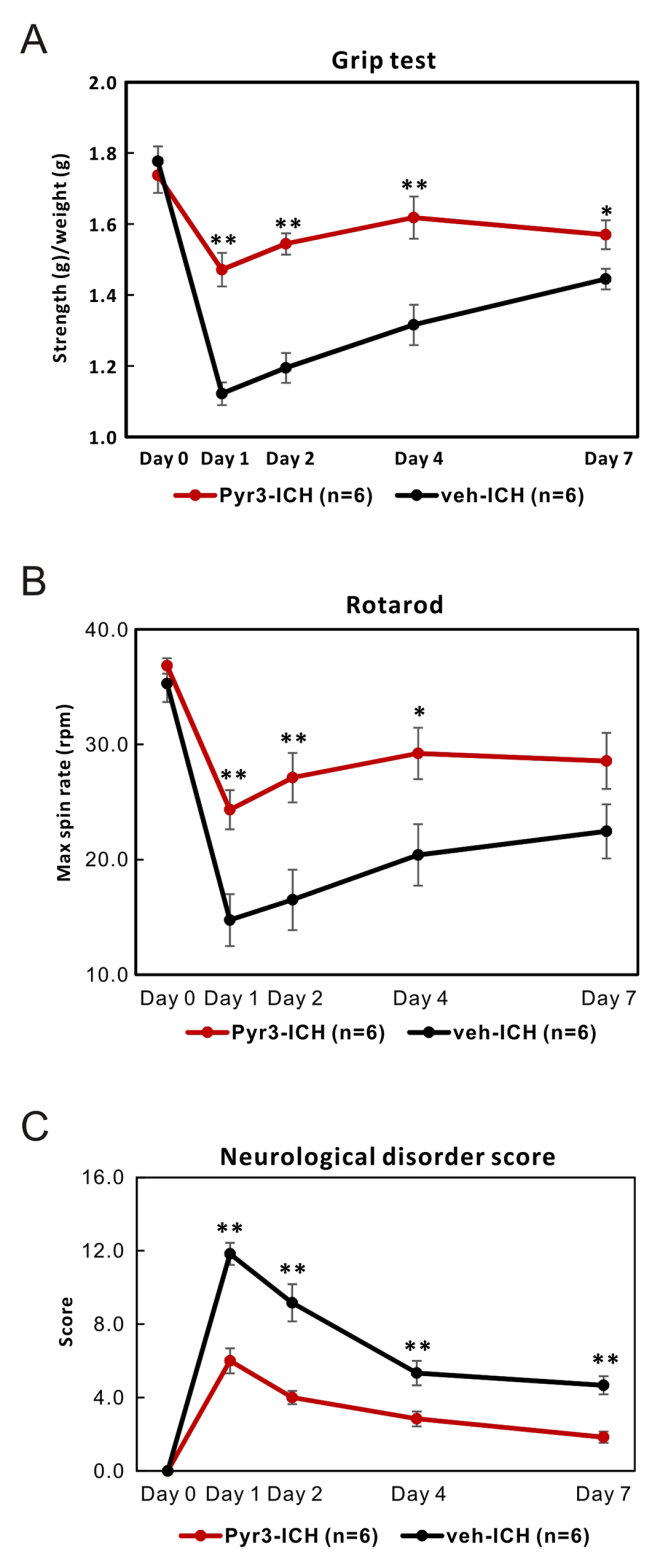
Pyr3 treatment significantly improves the neurobehavioral outcomes of ICH rats **(A)** Grip strength test was shown. **(B)** Rotarod test was shown. **(C)** Neurological disorder scores of ICH rats in both groups were recorded and plotted. Data are presented as the mean ± SEM. ^*^p < 0.05; ^**^p < 0.01; ^***^p < 0.001.

The neurobehavioral function tests was quantified and shown in Figure 4. The grip strength of ICH rats in Pyr3-treated group were significantly restored at 1, 2, 4, and 7 days after ICH induction (Figure 4A; Student’s *t* tests: *p* < 0.01 at day 1, 2, and 4, *p* < 0.05 at day 7 for vehicle-treated vs. Pyr3-treated groups).

The mean spin rates of Rodarod test of ICH rats in Pyr3-treated group were also significantly recovered at 1, 2, 4, and 7 days after ICH induction (Figure 4B; Student’s *t* tests: *p* < 0.01 at day 1 and day 2, *p* < 0.05 at day 4 for vehicle-treated vs. Pyr3-treated groups). In addition, neurological disorder scores in Pyr3-treated ICH rats were significantly less than vehicle-treated ICH rats (Figure 4C; Student’s *t* tests: all *p* < 0.01 for vehicle-treated vs. Pyr3-treated groups). The overall performance of ICH rats treating with Pyr3 significantly was better than vehicle-treated animals.

### Inhibition astrocytic activity also retards the progress at hyperacute and acute stages in autologous blood-induced ICH model

In order to confirm that the findings in collagenase-induced ICH could be also shown in another widely used ICH model, autologous blood-induced ICH model was established. Vehicle- or Pyr3-treated autologous blood-induce ICH rats were underwent the MRI assessments and GFAP immunohistochemistry. The results are illustrated in the Supplementary Materials.

In the Pyr3-treated group, the size of the hematoma was significantly decreased than vehicle-treated group at 12 h, 15 h, and 21 h after ICH induction (Supplementary Figure 1A and 1B; Student’s *t* tests: all *p* < 0.05 for vehicle-treated vs. Pyr3-treated groups). Pyr3 treatmentreduced the K^trans^ signals in the Pyr3-treated groups at 3 h after ICH induction compared with the vehicle-treated groups (Supplementary Figure 1A and 1C; Student’s *t* tests: *p* < 0.05 for vehicle-treated vs. Pyr3-treated groups). The perihematomal accumulation of astrocytes in the vehicle- and Pyr3-treated ICH rats at 0.5, 3, 12, 15, and 21 h after ICH induction was further examined using GFAP staining (Supplementary Figure 1D). The GFAP-positive areas were quantified and plotted (Supplementary Figure 1E). After Pyr3 treatment, the number of accumulated GFAP-positive astrocytes decreased at 3 h, 12 h, and 15 h after ICH induction (Supplementary Figure 1E). These results suggest collagenase is independent with the effects of Pyr3 and the beneficial effects of astroglial inhibition were both observed in collagenase-induced and autologous blood-induced ICH models.

## DISCUSSION

This is the first *in vivo* study to meticulously characterize the temporal evolution from the hyperacute (within 3 h) to acute stages (3–24 h) of ICH. Astrocytes act as the major cell population within the first 24 h of ICH rather than microglia or macrophages. Astrocyte activity was decreased using Pyr3; the activated astrocytes affected the lesion size, hematoma resolution, and BBB destruction. These findings support the notion that reactive astrocytes play a crucial role in the hyperacute and acute stages of ICH.

Although the most prominent deteriorations occur within the first day of ICH onset, the understanding of the pathogenic development of ICH during this period remains limited. Through MRI, *in vivo* changes in hematomas and edemas have been documented previously at various time points (1, 4, and 7 days after ICH onset) [25]. In the present study, we further characterized the MR features of hematoma within the first 24 h. The combination of T2WI and DWI is sensitive for differentiating injuries in hemorrhages, vasogenic lesions, and cytotoxic edema. Hemorrhage is associated with low T2WI and DWI signals at the lesion site [26]. Lesions with high or pseudonormal DWI and high T2WI signals are recognized as cytotoxic edema [27]. In hyperacute ICH, the lesions with low signals in both T2WI and DWI indicate a hemorrhage. In acute ICH, the lesions become hyperintense in T2WI and DWI, suggesting cytotoxic edema. As illustrated in our findings, hematoma during the first 24 h can be divided into hemorrhagic and cytotoxic edema phases according to the hyperacute and acute stages. These results imply that the initial management of ICH may be directed toward controlling hematoma growth and decreasing the hazards of hemorrhage such as iron toxicity. When ICH advances to the acute stage, appropriate treatment strategies should focus on BBB protection, edema attenuation, keep cerebral autoregulation, and homeostasis maintenance.

Increasing evidence of the crucial role of inflammation in ICH pathology is available; however, little is known about the cellular responses within the first 24 h after ICH onset. In the first 3 days after ICH onset, activated microglia and astrocytes form glial scars surrounding the hematoma [6]. In addition, the recruited macrophages infiltrate into perihematomal tissues from the third day until several weeks after ICH onset [6, 28]. However, there is insufficient research focusing on the pathogenesis occurring within the first day after ICH onset. Notably, numerous GFAP^+^ astrocytes were found in perihematomal regions within 24 h after onset, but only a few Iba1^+^ microglia or macrophages. Astrocytes possibly have a higher tolerance to oxidative stress and iron toxicity than other CNS cells such as neurons, microglia, and macrophages [7, 28]. In addition, during cerebral hemorrhaging, blood-derived factors such as thrombin may serve as a potential inducer of astrocytic proliferation and activation [29, 30]. These results demonstrate that astrocytes, rather than microglia or macrophages, are the most capable and dominant immune cells closely involved in the pathogenesis of the hyperacute and acute stages of ICH.

Reactive astrocytes are postulated to have dual roles following CNS injuries including ICH [7]. The protective effects could be provided by repair of BBB, prevention of glutamate toxicity, tolerance of oxidative stress, or secretion of neuroprotective factors [31-33], while potentially detrimental effects might be caused by the release of proinflammatory cytokines such as IL-1 β and TNF- α [11] and reactive oxygen species production [12]. Wang et al demonstrated that astrocytic heme oxygenase-1 exacerbates early brain injury after ICH [16]. Recently, the reactive astrocytes were also implicated in cytotoxic edema development via upregulation of astrocytic water channel aquaporin-4 [34]. These results indicate that activated astrocytes may play opposing roles following hemorrhagic injuries.

Pyr3, a suppressor of astrocytic activity, has been reported to attenuate reactive astrogliosis and ameliorate neurological deficits resulting from both collagenase and autologous blood infusion-induced ICH models [20, 21]. Thrombin is a predominant blood-derived factor that extravagates into the brain parenchyma during cerebral hemorrhaging and is crucial for astrocyte activation [29]. These responses are regulated by the G-protein-coupled receptor TRPC3, which is closely associated with calcium influx [20]. Although TRPC3 is expressed in many cell types, but the abundant TRPC3 receptors are expressed in CNS, especially in astrocytes. In previous study showed that Pyr3 can specifically inhibit the thrombin-TRPC3 signaling and diminish the astrocyte activity at various time points (1, 4, and 7 days after ICH onset) [20, 21]. These quoted studies indicated that Pyr3 reduced the perihematomal accumulation of astrocytes and ameliorated ICH-induced brain secondary injury. However, the inhibitory effects of Pyr3 against ICH and astrocyte activity with the first 24 h remain unknown. In present study, we used Pyr3 to modulate the activity of astrocytes for disclosing their roles during the first 24 h of ICH. We found that astrocytic accumulation was peaked at 12 h after ICH, whereas significantly fewer Iba1-positive MMΦ were observed in the perihematomal regions at all time points, indicating that activated astrocytes seems playing a more crucial role in the pathogenic process of hyperacute and acute stages of ICH.

**Figure 5:**
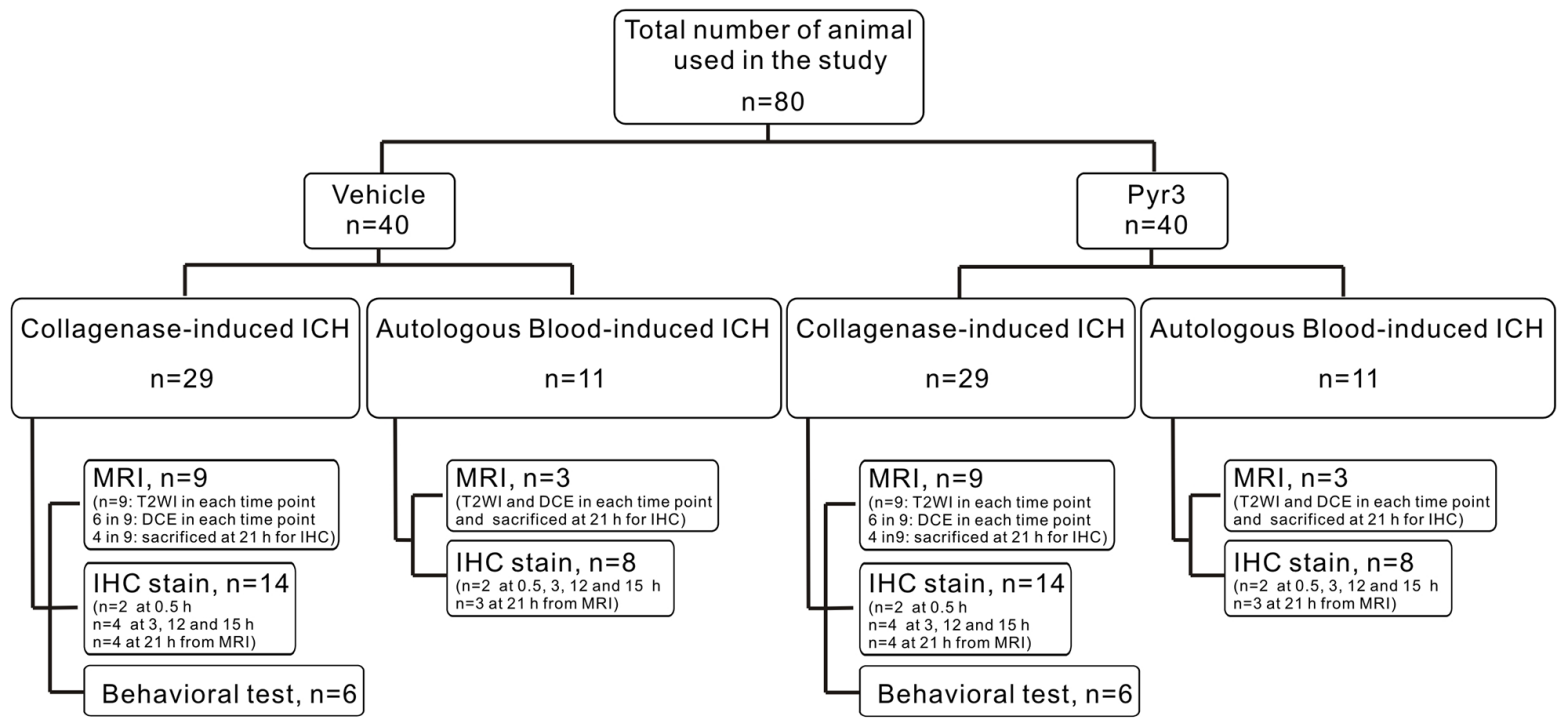
Flow chart of experimental design

By suppressing astrocyte activity via Pyr3, we demonstrated the critical role of astrocytes in the pathological evolution of hematoma within 24 h of ICH induction. In order to confirm that if these findings were only found in collagenase-induced model and to prevent the results may be interfered by the influence of Pyr3 to collagenase, the autologous blood-induced ICH model was established. Autologous blood-induced ICH rats received the vehicle or Pyr3 treatments were underwent MRI assessments and histological confirmation. As the results in blood injected ICH rats shown in Figure 5, these findings indicate that inhibition of astrocytic activity by Pry3 alleviates the sequela and improve the overall outcomes in hyperacute and acute stages of both rodent ICH models.

ICH is often associated with changes in BBB permeability. When brain-resident astrocytes surround hemorrhagic lesions, these reactive astrocytes have been shown to promote BBB disruption, resulting in brain edema formation [14, 35, 36] through the secretion of proinflammatory factors such as IL-1β, TNF-α [28, 37], and matrix metalloproteinases (MMPs) [14, 38]. K^trans^ maps illustrate that severe BBB breakdown occurs in the initial hours and the degree of BBB destruction decreases over time from the hyperacute to acute stages. The activity of astrocytes triggered by blood-derived factors may increase, and subsequently, these astrocytes may secrete deleterious factors to disrupt the BBB integrity. This scenario was confirmed in the present study by the detrimental effect of astrocytes on BBB destruction diminishing over time once the astrocytic activity was suppressed using Pyr3. According to our previous study [25], although astrocytic AQP-4 expression was downregulated by hyperglycemia, it increased the vasogenic edema and severity of BBB destruction in ICH. These findings support the idea that astrocytes play a key role in the pathological development of ICH and BBB dysfunction. And the suppression of the activity of astrocytes in hyperacute and acute stage of ICH may offer an alternative thinking to keep BBB integrity.

When BBB disruption occurs in ICH, iron-containing components such as hemoglobin are released into the brain parenchyma. With the increase in the release of concentrated iron-containing components, the homeostatic mechanisms of iron metabolism become decompensated, and the excess iron produces significant toxicity and causes neuronal death [39]. To cope with the hemorrhagic brain injury, activated astrocytes exhibit higher tolerance to iron toxicity and express heme oxygenase 1 (HO-1) as the key enzyme of heme metabolism to support the functions of iron clearance [16, 17]. However, significant upregulation of astrocytic HO-1 expression was reported to exacerbate early brain injury after ICH onset [16]. Consistent with our findings, suppressing astrocytic activity using Pyr3 is crucial for lesion size control, prolonging the hemorrhagic phase, and postponing the course of hemoresolution. Therefore, we suggest that appropriate control the activity of astrocytes in ICH may potentially achieve hematoma size reduction, BBB protection, and less mass effect, which are substantially more critical than early hemoresolution, particularly within the decisive first 24 h after onset. In other words, a gentler course of hemoresolution may prevent severe immune agitation, which may induce more severe secondary brain injuries in the hyperacute and acute phases of ICH.

Recent studies indicated astrocyte-producing chemo-attracting factors may participate in CNS disease progression and inflammation [40, 41]. Monocyte chemoattractant protein-1 (CCL2), for instance, induces monocyte infiltration and BBB compromise based on CCL2/CCR2 signaling system in several CNS diseases [42, 43]. This process is exacerbating in stroke, especially in ICH, because CCL2 can exist in more active truncated form under the digestion of plasma plasmin [44]. In other words, the higher GFAP-expressing astrocytes population may produce more original CCL2 in acute ICH and start a vicious cycle. The CCL2 knockout mice with ICH had less lesion size in acute stage but showed poorer repairing velocity [42]. This can echo to our MRI results, of which astrocytes inhibition reduced hematoma expansion but slowed down the process of hemo-resolution.

ICH is a devastating disease with no definite promising therapy. The lack of available acute therapeutic options for ICH may be because of insufficient background knowledge regarding this disease. A clear delineation of the multiple phases of ICH and identification of the critical risk factors in the pathogenesis are essential for developing optimal therapeutic strategies. According to preclinical and clinical studies in the past decade, successful treatment of ICH will most likely be multifaceted. A personalized therapeutic protocol that can be dynamically adjusted according to different conditions may finally lead to successful treatment [45].

In summary, via the suppression of astrocytic activity, both models of collagenase-induced and autologous blood-induced ICH rats exhibited decreased size of hematoma expansion, less BBB destruction, reduced astrocytic accumulation in perihematomal regions (Supplementary Figure 2) and gain better outcomes. In the present study, we first demonstrated that there are two phases of ICH within the first 24 h: hemorrhagic and cytotoxic edema phases, implying that the specific treatments applied to these phases of ICH should be different. The accurate diagnosis for differentiating the different phases of pathological processes of ICH may assist in selecting appropriate treatments for ICH patient management. Second, by addressing the significance of activated astrocytes on the first day of ICH, we suggest that astrocytes have a key role in the hyperacute and acute stages of ICH. They may be directly involved in all major destructive and constructive activities of ICH, particularly during the first 24 h. Suppression of astrocytic activity in the hyperacute stage may minimize the effects of astrocyte-induced inflammation and improve overall outcomes.

## MATERIALS AND METHODS

### ICH rat models and Pyr3 treatment

All animal experimental protocols were in compliance with the regulations of the Animal Welfare Committees of Academia Sinica, Taipei, Taiwan, and were performed in accordance with the guidelines of the Animal Welfare Protection Act of the Department of Agriculture, Executive Yuan in Taiwan. All of experiments in this study followed the Animal Research Reporting *in vivo* Experiments (ARRIVE) guidelines. The rats were housed in a controlled environment and triple-housed in plastic cages with access to food and water ad libitum. The 9-week-old male Sprague–Dawley rats with body weight between 250-300 grams were purchased from the National Laboratory Animal Center of Taiwan.

Experimental collagenase-induced ICH model was described previously [21]. In order to confirm the effectiveness by another widely used rodent ICH model, autologous blood-induced ICH model [46], the 30μL of fresh blood taken from the tail vein within 60 secs was infused into the striatum over 10 mins. To suppress astrocyte activity [20, 21], Pyr3, a TRPC3 inhibitor, was suspended in 10% PEG-45 hydrogenated castor oil at a concentration of 4 mmol/L and intraventricularly administered into the lateral ventricle ipsilateral to the site of lesions (bregma,–0.5 mm; lateral, –1.5 mm or 1.5 mm; depth, 3.5 mm) at a concentration of 0.2 mmol/5 μL, 10 min after the ICH induction. All experiments and their subsequent evaluations were performed by an experimenter blinded to the identity of groups.

A total of 80 rats were used in this study, and the experiment design was showed in Figure 5. The collagenase-induced ICH rats were randomly assigned to receive a vehicle alone (phosphate-buffered saline, PBS) or Pyr3. A total of 18 rats were assigned to the MRI experiments for T2-weighted imaging (T2WI) and diffusion-weighted imaging (DWI) acquisition (*n* = 9 each group; total, *n* = 18). In each group, six rats were selected for additional dynamic contrast-enhanced (DCE) MRI experiments at 3, 15, and 21 h after ICH induction. After the last time point, four vehicle-treated and four Pyr3-treated ICH rats were immediately sacrificed for 21-h immunohistochemistry. An additional 28 rats (*n* = 2 for 0.5-h, *n* = 4 for 3-h, *n* = 4 for 12-h, and *n* = 4 for 15-h immunohistochemistry; *n* = 14 in each group; total, *n* = 28) were sacrificed for immunohistochemistry at 0.5, 3, 12, and 15 h after ICH induction. To verify neurobehavioral function, the 12 rats (*n*=6 in each group) underwent bilateral grip tests, Rotarod performance tests, and neurological disease scoring (NDS). The effective sample size in each group was calculated based on the “resource equation” method proposed by Festing et al [47, 48] and followed the guidelines proposed by Charan and Kantharia [49]. Furthermore, 22 rats (*n*=3 per group for MRI assessments and for 21-h immunohistochemistry, total *n* = 6; *n* = 2 per group at 0.5 h, 3 h, 12 h and 15 h for immunohistochemistry, total *n* = 16) were induced by the tail vein blood for confirmation.

### Magnetic resonance imaging methods

The rats were examined through MRI at 1, 3, 12, 15, and 21 h after injection by using a horizontal 7.0-T spectrometer (PharmaScan 70/16, Bruker, Germany) with an active shielding gradient of 300 mT/m in 80 μs. Images were acquired using a 72-mm birdcage transmitter coil and a separate quadratic surface coil for signal detection. Each rat was initially anesthetized with 5% isoflurane flowing in O_2_ at 2 L/min and then maintained at 1.5%–2.0%. The breathing rate was maintained at 60–70 breaths/min. T2WI images were acquired using a fast spin-echo sequence with the following parameters: field of view (FOV) = 2.56 cm^2^, slice thickness = 1 mm, number of slices = 8, repetition time (TR)/echo time (TE) = 3000/70 ms, echo train length = 8, number of excitations (NEX) = 12, and matrix size = 256 × 128 (zero filled to 256 × 256). DWI images were obtained using the Stejskal–Tanner spin-echo sequence (TR/TE = 2600/35 ms, NEX = 1, diffusion gradient duration/separation = 5/12 ms, two b values = 0 and 1100 mm^2^/s applied along the X-direction). To reduce the potential for gadolinium (Gd; Gadoevist, AG, Germany) accumulation, DCE MRI was performed only at 3, 15, and 21 h after injection. T2WI, DWI, and DCE MRI were acquired at the same location. DCE MRI was performed using a dynamic series of 80 T1-weighted gradient-echo images (TR/TE = 130/4 ms, flip angle = 30°, number of slices = 8, FOV = 2.56 cm^2^, matrix size = 128 × 128, slice thickness = 1 mm, and NEX = 1). An intravenous bolus injection of 0.2 mmol/kg Gd was administered during acquisition of the eighth frame. The contour of the lesion delineation, volume measurement, and K^trans^ map calculation were analyzed according to a previous study [50]. The contour of the lesions was delineated based on the contrast provided by the T2WIs between lesions and brain tissues. Volume was calculated by summing the area in three dimensions using Avizo software (version 6.0, Visualization Sciences Group, MA, USA). In DCE-MRI, the kinetic analysis of dynamic signal enhancement by Gd-DTPA was based on the compartment model of Tofts [51]. Among Tofts model, the K^trans^ is sensitive to the regions with higher vascular permeability, which is closely associated with the leakage of BBB [52].

### Neurobehavioral testing

ICH rats from PBS-treated and Pyr3-treated groups were selected and subjected to test neurobehavioral functions (n=6, each group). The bilateral grip test, Rotarod performance test, and neurological disease scoring (NDS) were used to measure neurological function before and on 1, 2, and 4 days after ICH [25]. Briefly, the grip strength test measured the strength (in g) to hold onto a steel grip-bar (IMADA digital force gauge, Model DPS-5R: range 0–5 kgf) with forepaws. Tests were conducted by gently and quickly setting a rat’s paws on the grip bar, and the animal was pulled horizontally away from the bar until the rat releases its grip. The performance was evaluated by an observer blinded to the treatment group and the identity of the rats. Grip strength was normalized to body weight. To assay the motor abilities of each experimental group, rats were conditioned on the Rotarod cylinder (TSE technical & scientific 337500), and the duration for which the rats remain on the Rota rod was recorded. For each individual rat, three trials were performed with a 15-minute break. Animals were trained in advance at three separate days before surgery. In addition to the grip strength and Rodarod tests, rats treated with vehicle or Pyr3 are evaluated for their degree of neurological deficits (NDS), a maximal 28-point system. An investigator blinded to the experimental cohort scores all rats on six neurologic tests, including body symmetry, gait, climbing, circling behavior, front limb symmetry, compulsory circling, and whisker response. Each test was scored from 0 for normal up to 4 with increasing severity.

### Histological examination

The rats were perfused at 0.5, 3, 12, 15, or 21 h after intrastriatal collagenase injection by using saline followed by 4% paraformaldehyde. The brains were removed and embedded in paraffin. Five-micron-thick brain sections were subjected to immunofluorescence staining of GFAP (1:2000; Dako) and Iba1 (1:400; Wako) by following a previously described method [50]. Four rats in each vehicle- and Pyr3-treated group at each time point were selected for quantification. Four representative images (at 40× magnification) were selected from each lesion, and the coverage areas and cell numbers were analyzed using ImageJ software. The boxes 1 represent the four preselected regions of the striatum (Figure 1, panel A).

### Statistical analysis

The results obtained from independent experiments are presented as the mean ± SEM. Between-groups differences were tested using analysis of variance (ANOVA) and Fisher’s post hoc tests. Student’s two-tailed *t* test was used to determine the difference in the means between any two groups. All statistical analyses were performed using StatView software (SAS Institute, NC, USA). Results with *p* <0.05 were considered statistically significant.

## SUPPLEMENTARY MATERIALS FIGURES



## Abbreviation

ICH: intracerebral hemorrhage
MMΦ: microglia/macrophages
BBB: blood–brain barrier
GFAP: Glial fibrillary acidic protein
CNS: central nervous system
TRPC3: transient receptor potential canonical 3
MRI: Magnetic resonance imaging
T2WI: T2-weighted imaging
DWI: diffusion-weighted imaging
NDS: neurological disease scoring
Pyr3: Ethyl-1-(4-(2,3,3-trichloroacrylamide)phenyl)-5-(trifluoromethyl)-1H-pyrazole-4-carboxylate
PBS: phosphate-buffered saline
