# Supplementary material for: Inhibition of astrocytic activity alleviates sequela in acute stages of intracerebral hemorrhage
Source: Oncotarget. 2017 Oct 24;8(55):94850–61. doi: 10.18632/oncotarget.22022 (PMC5706917; doi:10.18632/oncotarget.22022)
Supplement: Supplementary file 1 [file oncotarget-08-94850-s001.pdf]

Inhibition of astrocytic activity alleviates sequela in acute stages of intracerebral hemorrhage

SUPPLEMENTARY MATERIALS

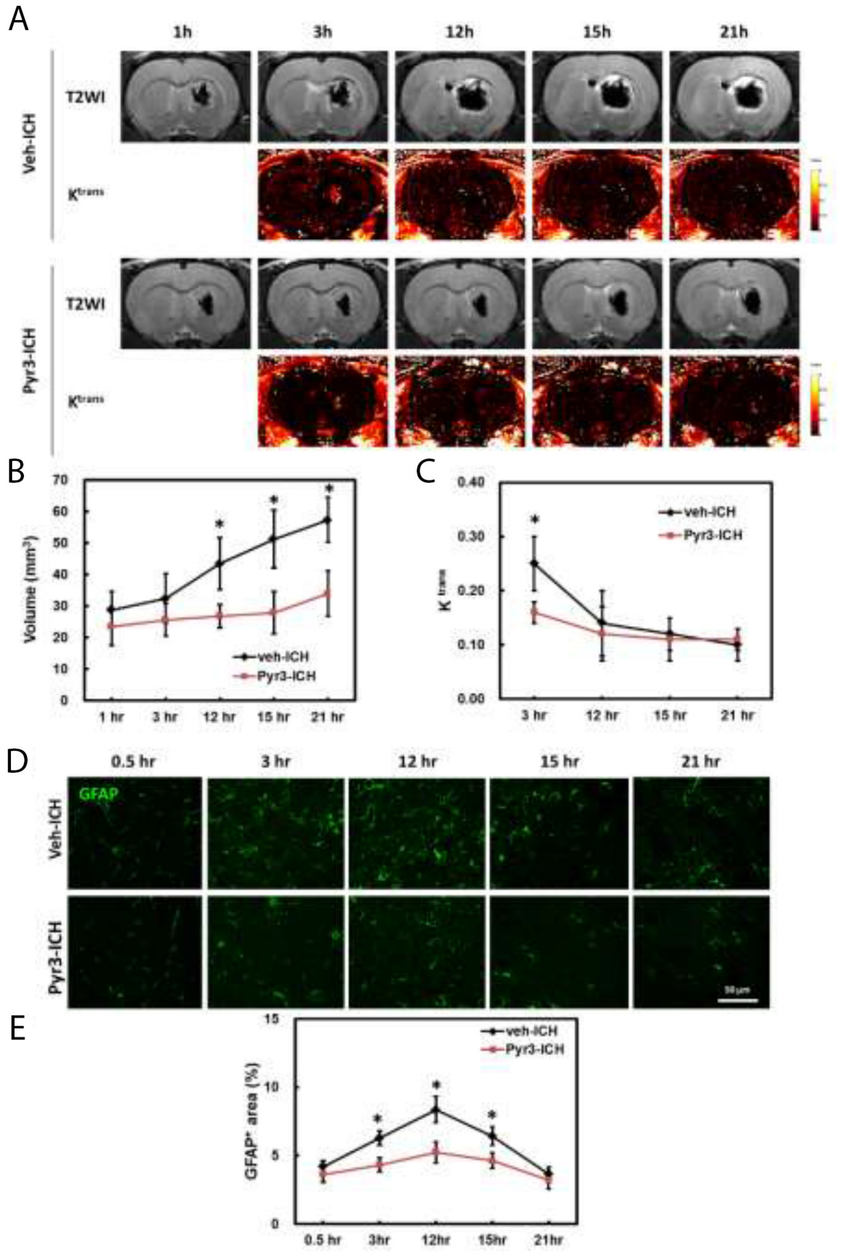

Supplementary Figure 1: Inhibition astrocytic activity also retards the progress at hyperacute and acute stages in autologous blood-induced ICH model. **(A)** Representative T2WI and K^{trans} maps of vehicle- and Pyr3-treated rats in autologous blood-induced ICH model. **(B)** Volumes of the hematomas at different time points were estimated using T2WIs. **(C)** The graph of the K^{trans} values. **(D)** Representative GFAP staining of vehicle- and Pyr3-treated rats. **(E)** GFAP staining of vehicle- and Pyr3-treated groups plot. Data are presented as the mean ± SD. $^{*}p < 0.05$.

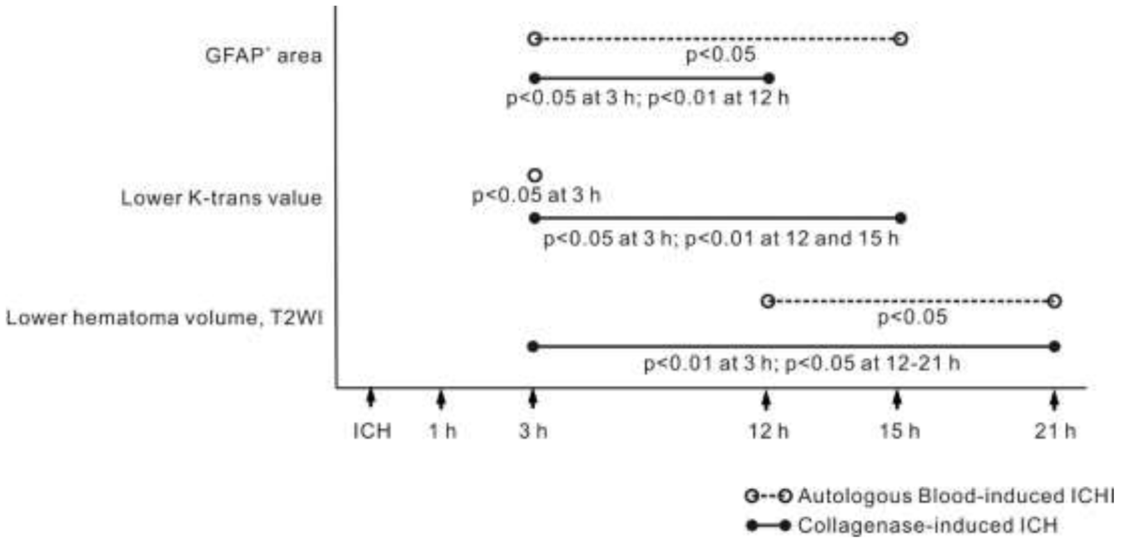

Supplementary Figure 2: Finishing chart for statistical significance of each assay. All collected data was compared between Pyr-3-ICH groups with Veh-ICH groups.
